# Thank you to our reviewers of 2022

**DOI:** 10.1002/epi4.12700

**Published:** 2023-02-17

The Editors of *Epilepsia Open* are very grateful to our Associate Editors and editorial board members as well as the following individuals for reviewing manuscripts in the past year. We are deeply appreciative of their insight, time and effort.

Aristea S. Galanopoulou

Editor‐in‐Chief, *Epilepsia Open*

Dong Zhou

Deputy Editor, *Epilepsia Open*

*1‐2 reviews, **≥3 reviews

Eishi Asano**

Stéphane Auvin**

Sara Baldassari**

Emanuele Bartolini**

Sándor Beniczky**

Sonu Bhaskar**

Francesca Bisulli**

Jeffrey Buchhalter**

Giovanni Falcicchio**

Guilherme Fialho**

Taha Gholipour**

Mubeen Janmohamed**

Hoon‐Chul Kang**

Karl Klein**

Barbara Kroner**

Naoto Kuroda**

Hyo Lee**

Rosa Michaelis**

Solomon Moshé**

Jie Mu**

Kenneth Myers**

Rima Nabbout**

Heath Pardoe**

Anup Patel**

Martina Perinelli**

Piero Perucca**

Jan Remi**

Harvey Sarnat**

Adam Strzelczyk**

Kette Valente**

Alberto Verrotti di Pianella**

Michael Wong**

Gaetano Zaccara**

Taylor Abel*

Sami Aboumatar*

Naoto Adachi*

Patrick Adjei*

Timothy Ainger*

Naoki Akamatsu*

Cigdem Akman*

Dongmei An*

Amir Aschner*

Jerome Aupy*

Selma Aybek*

Jacquelyn Bainbridge*

Simona Balestrini*

Melissa Barker‐Haliski*

Gregory Bergey*

Salvatore Berlangieri*

Federica Bertaso*

Leonilda Bilo*

Valeri Borger*

Anatol Bragin*

Francesco Brancati*

Benjamin Brinkmann*

Giacomo Brisca*

Andreas Brunklaus*

Ralph Buchert*

Lorenzo Caciagli*

Laura Canafoglia*

Giuliana Cangemi*

Roberto Caraballo*

Esper Cavalheiro*

Emanuele Cerulli Irelli*

Felix Chan*

Lynn Chapieski*

Chien Chen*

Zhibin Chen*

Zhuying Chen*

Michael Chez*

Yotin Chinvarun*

Honor Coleman*

Luis Concha*

Giorgia Conte*

Antonietta Coppola*

Duccio Maria Cordelli*

Daniel Costello*

Douglas Crompton*

Claudia Cuccurullo*

Arthur Cukiert*

Scott Demarest*

Antoine Depaulis*

Carlo Di Bonaventura*

Yan Dong*

Elizabeth Donner*

Gianluca D'Onofrio*

Christian Dorfer*

Mattias Duempelmann*

John Duncan*

Roderick Duncan*

John Dunne*

Maurizio Elia*

Colin Ellis*

Christin Eltze*

Andrew Escayg*

Firas Fahoum*

Edoardo Ferlazzo*

Carolina Ferreira‐Atuesta*

Robert Fisher*

Patrick Forcelli*

Jacqueline French*

Daniel Friedman*

Denson Fujikawa*

Ayataka Fujimoto*

Hisako Fujiwara*

Norberto Garcia‐Cairasco*

Elena Gardella*

Steve Gibbs*

Barry Gidal*

Frank Gilliam*

Filippo Giorgi*

Beatriz Giraldez*

Loretta Giuliano*

Joseph Glykys*

Vadym Gnatkovsky*

Teneille Gofton*

Daniel Goldenholz*

Marco Gonzalez*

Jean Gotman*

Zachary Grinspan*

Cecil Hahn*

Hajo Hamer*

Stephen Hantus*

David Henshall*

Michael Hildebrand*

Martin Holtkamp*

George Ibrahim*

Jean Isnard*

Naoum Issa*

Yosuke Ito*

Damir Janigro*

Lin‐Hua Jiang*

Cecilie Johannessen Landmark*

Nigel Jones*

Shilpa Kadam*

Andres Kanner*

Jaideep Kapur*

Sunil Karande*

Jennifer Kearney*

Ching Soong Khoo*

Kitsarawut Khuancharee*

Gerhard Kluger*

Eliane Kobayashi*

Christian Korff*

Sanjeev Kothare*

Stjepana Kovac*

Izumi Kuramochi*

Churl‐Su Kwon*

Sylvain Laborde*

Lieven Lagae*

Simona Lattanzi*

Wang‐Tso Lee*

Young‐Mock Lee*

Johannes Lemke*

Massimo Leone*

Costin Leu*

Laura Librizzi*

Ling Liu*

Lili Long*

Maria‐Leonor Lopez Meraz*

Aimee Luat *

Heiko Luhmann*

Lucille Lumley*

Pavel Mares*

Andrey Mazarati*

David McArthur*

Oriano Mecarelli*

Marco Medina*

Stefano Meletti*

Cameron Metcalf*

Alicja Mryzyglod*

Angelika Mühlebner*

John Mytinger*

Hiroki Nariai*

Jun Natsume*

Maromi Nei*

Aidan Neligan*

Duong Nhu*

Andreea Nissenkorn*

Giulia Nobile*

Francesco Noe*

Jan Novy*

Hirokazu Oguni*

Antonio Oliva*

Filiz Onat*

Tomonori Ono*

Sandra Orozco‐Suarez*

Laila Ouchen*

Eleonora Palma*

Justyna Paprocka*

Jun Park*

Liborio Parrino*

Archana Patel*

Victor Patterson*

Eric Payne*

Bartholomew Pederson*

Patricia Penovich*

Quentin Pittman*

Viola Podewils*

Pierre‐Marie Preux*

Michael Privitera*

Federica Provini*

Jan Pukropski*

Mark Quigg*

Ramya Raghupathi*

Sridharan Ramaratnam*

Janir Ramos Da Cruz *

François Rassendren*

Genevieve Rayner*

Colin Reilly*

Jong Rho*

George Richerson*

Kate Riney*

Aleksandar Ristic*

Antonella Riva*

Roberta Roberti*

Luisa Rocha*

Jillian Rosengard*

Theodor Rüber*

Emilio Russo*

Pauline Samia*

Daniel San‐Juan*

Raman Sankar*

Rani Sarkis*

Shifteh Sattar*

Tyson Sawchuk*

Morris Scantlebury*

Mariasavina Severino*

Masud Seyal*

Aashit Shah*

Hiroshi Shigeto*

Jerry Shih*

Hideaki Shiraishi*

Pallop Siewchaisakul*

Hugh Simpson*

Gagandeep Singh*

Mary Lou Smith*

Daichi Sone*

Guenther Sperk*

Carl Stafstrom*

Remi Stevelink*

Pasquale Striano*

Joseph Sullivan*

Hrishikesh Suresh*

Rainer Surges*

Olafur Sveinsson*

Jerzy Szaflarski*

Pierre Szepetowski*

William Szurhaj*

Christopher Tailby*

James Tao*

Jeffrey Tenney*

William Theodore*

Roland Thijs*

Maria Thom*

Mario Tombini*

Torbjörn Tomson*

Joseph Tracy*

Meng‐Han Tsai*

Riyo Ueda*

Lata Vadlamudi*

Andreas van Baalen*

Giulia Varotto*

Jana Veliskova*

Liana Veneziano*

Lawrence Ver Hoef*

Vicente Villanueva*

Lucy Vivash*

Tim von Oertzen*

Felix von Podewils*

Sarah von Spiczak*

Randi von Wrede*

Bernd Vorderwülbecke*

Rob Voskuyl*

David Vossler*

Genna Waldman*

Mengyang Wang *

Christopher Whalley*

Karen Wilcox*

Elaine Wirrell*

Juri‐Alexander Witt*

Markus Wolff*

Stefan Wolking*

Joseph Yaria*

Sara Zagaglia*

Rina Zelmann*

Gang Zhang*

Maryam Zoghi*

Georg Zoidl*

